# How to Differentiate Gout, Calcium Pyrophosphate Deposition Disease, and Osteoarthritis Using Just Four Clinical Parameters

**DOI:** 10.3390/diagnostics11060924

**Published:** 2021-05-21

**Authors:** Dmitrij Kravchenko, Charlotte Behning, Raoul Bergner, Valentin Sebastian Schäfer

**Affiliations:** 1 Department of Diagnostic and Interventional Radiology, University Hospital of Bonn, 53127 Bonn, Germany; dmitrij.kravchenko@ukbonn.de; 2 Institute for Medical Biometrics, Informatics and Epidemiology (IMBIE), University Hospital of Bonn, 53127 Bonn, Germany; behning@imbie.meb.uni-bonn.de; 3 Medical Clinic A: Clinic for Internal Medicine, Hematology, Nephrology, Infektiology and Rheumatology, Klinikum Ludwigshafen, 67063 Ludwigshafen, Germany; BERGNERR@klilu.de; 4 Clinic of Internal Medicine III, Hematology, Oncology, Rheumatology and Clinical Immunology, University Hospital of Bonn, 53127 Bonn, Germany

**Keywords:** gout, CPPD, osteoarthritis, diagnosis, ultrasound

## Abstract

Clinical differentiation between gout, osteoarthritis (OA), and calcium pyrophosphate deposition disease (CPPD) remains a hurdle in daily practice without imaging or arthrocentesis. We performed a retrospective analysis of consecutive patients with gout, CPPD, and OA at a tertiary rheumatology center. A total of 277 patients were enrolled, with 164 suffering from gout, 76 from CPPD, and 37 from OA. We used ANOVA and conditional inference tree analysis (Ctrees) to find associations between clinical, laboratory, and imaging data and gout, OA, and CPPD. The sonographic double contour sign was unable to differentiate gout from CPPD. Ctrees were able to exclude OA and CPPD as possible differentials based on elevated uric acid, C-reactive protein (CRP), presence of arterial hypertension, and sex, diagnosing gout with a sensitivity and specificity of 95.1% and 41.6%, respectively. Elevated CRP was observed using simple linear regressions in patients with type II diabetes, higher cumulative joint scores, increased number of affected joints, as well as elevated uric acid, erythrocyte sedimentation rate, and leukocyte count. Ctrees were able to differentiate gout, OA, and CPPD based on just four characteristics. Inflammatory response correlated with type II diabetes, more or larger joint involvement, and elevated uric acid levels.

## 1. Introduction

The differentiation between inflammatory osteoarthritis (OA), the most common type of arthropathy, and gout, the most prevalent crystal arthropathy, is often difficult in daily clinical practice without laboratory tests, imaging, or arthrocentesis [1]. To further complicate matters, calcium pyrophosphate deposition disease (CPPD) can manifest with similar symptoms and an overlapping joint pattern.

Osteoarthritis is commonly defined as a heterogeneous group of conditions that lead to arthralgia [2]. Diagnostic criteria are divided into clinically defined OA and radiographically defined OA. Clinical diagnostic criteria are based on symptoms and signs on physical examination, while radiographically defined OA relies on the Kellgren–Lawrence scale [3]. This scale classifies OA according to formation of osteophytes, periarticular ossicles, narrowing of joint space associated with sclerosis of subchondral bone, small pseudocystic areas with sclerotic walls, and altered shape of bone ends [4]. Ultrasound allows the detection of a wide spectrum of pathologic findings indicative of OA, involving articular cartilage, bony cortex, and synovial tissue [5].

The etiology of gout is poorly understood. Simplified, the phagocytosis of monosodium urate crystals by leukocytes in joints and soft tissues results in an inflammatory cascade [6]. Acute attacks may then be provoked by specific triggers, such as excessive alcohol or meat consumption. 

CPPD is thought to occur when an imbalance in inorganic pyrophosphate production and pyrophosphatase leads to saturation and precipitation of CPP crystals near the surface of cartilage [7,8]. If these precipitated crystals do not cause any symptoms but are visualized, for example, on radiographs, they are termed as chondrocalcinosis. CPPD was agreed as an umbrella term reserved for acute arthritis caused by CPP crystals, OA with CPPD, and chronic CPP crystal arthritis by the European League Against Rheumatism (EULAR), but the terminology still remains inconsistent across studies [9].

The diagnostic reference standard for gout and CPPD remains arthrocentesis with subsequent crystal visualization under compensated polarized light microscopy as well as leukocyte cell count of the aspirate [6], while radiographs and clinical examination remain the reference standard for diagnosing OA [10]. Musculoskeletal ultrasound can aid in diagnosis of all three diseases via validated findings such as the double contour (DC) sign, aggregates or tophi and erosions in gout, intracartilaginous hyperechogenicities (iHE) in CPPD, and osteophytes in OA as disease hallmarks [5,9,11,12,13].

This study retrospectively analyzed clinical, ultrasound, and laboratory parameters of 277 consecutive patients suffering from gout (*n* = 164), OA (*n* = 37), or CPPD (*n* = 76). The goal of this study was to elucidate reliable prognostic biomarkers for any of the three arthropathies, which could aid in their differentiation when imaging or arthrocentesis are unavailable or outstanding. Secondary goals included observation of the inflammatory response based on C-reactive protein (CRP) in relation to other biomarkers.

## 2. Materials and Methods

### 2.1. Patients

All consecutive patients presenting with acute arthralgia of any joint and a diagnosis of gout, CPPD, or OA who sought treatment at the Department of Rheumatology at the Hospital Ludwigshafen, Ludwigshafen, Germany, between 2014 and 2017 were included in this retrospective study. Patients diagnosed with more than one arthropathy were excluded from the final analysis. Analysis included routine laboratory values such as serum concentrations of ferritin, CRP, erythrocyte sedimentation rate (ESR), and serum uric acid. Patients must have had undergone routinely performed ultrasound of the affected joint with assessment regarding the degree of vascularization (DoV), the DC sign, and the iHE sign as defined below. In case of oligoarthritis, all joints underwent sonography but only the most painful joints were aspirated. Each patient must have also had routine arthrocentesis performed with subsequent compensated polarized light microscopy of the most affected joints and, if possible, leukocyte cell count of the joint aspirate. Additional patient characteristics such as sex, age, weight, height, body mass index (BMI), diabetic status, arterial hypertension, renal function, cumulative joint score, and number of affected joints were extracted. Current patient medication was not considered unless it was specific to gout, OA, or CPPD.

Patients were classified as hypertensive or diabetic if the condition had already been documented in previous medical reports. Renal status was classified according to the Kidney Disease Improving Global Outcome (K-DIGO) guidelines into stages of kidney disease based on the glomerular filtration rate (GFR) measured by the chronic kidney disease epidemiology collaboration (CKD-EPI) formula: 1 = GFR >90 mL/min, 2 = GFR 89–60 mL/min, 3 = GFR 59–30 mL/min, 4 = GFR 29–15 mL/min, and 5 = GFR <15 mL/min [14]. The cumulative joint score is an internally developed method to objectively quantify joint involvement and adjust for joint size. All affected joints were rated on a point system of 1 to 3 for each affected joint. One point was assigned to small joints such as the finger and toe joints. Two points were for middle-sized joints such as the ankle, elbow, or wrist. Three points were for large joints such as the hip, shoulder, or knee. A summary of the system used is presented in Table 1. For the number of affected joints, all affected joints, regardless of size, were added together and weighted equally.

### 2.2. Ultrasound Examination

**Figure 1:**
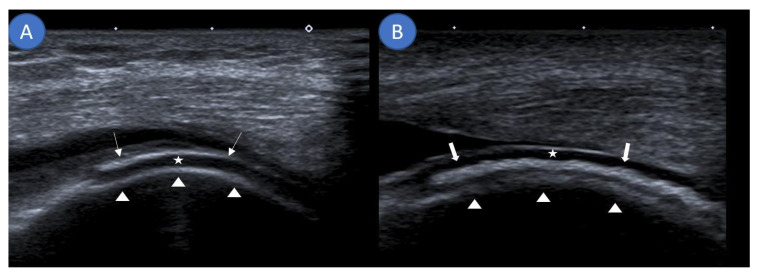
Differentiating the double contour sign from an intracartilaginous hyperechogenicity. (**A**) Sagittal ultrasound demonstrating monosodium urate deposits (arrows) as a hyperechoic band running parallel to the echo-free hyaline cartilage (star) at the suprapatellar knee joint. Together with the hyperechoic margin created by the femur (arrow heads), this forms the double contour sign. (**B**) Sagittal ultrasound along the lateral femoral condyle showing an intracartilaginous hyperechogenicity (thick arrows) visualizing calcium pyrophosphate deposits consistent with the intracartilaginous hyperechogenicity sign.

Musculoskeletal ultrasound examinations were performed by a board-certified musculoskeletal ultrasonographer with German Society for Ultrasound in Medicine (DEGUM) level III (highest level instructor) certification at the Clinic for Internal Medicine, Hematology, Nephrology, Infectiology and Rheumatology, Klinikum Ludwigshafen, Ludwigshafen, Germany, using an Aplio 400 Toshiba (Canon Medical Systems GmbH, Neuss, Germany) machine with linear or hockey stick transducers (5–14 MHz). Ultrasound examinations assessed for the DC sign and DoV as well as noted the presence of an iHE sign. The DC sign was defined as hyperechoic bands adjacent to hyaline cartilage (Figure 1A) [15]. If these hyperechoic signals could be confidently placed inside the cartilage and not on a tangent with it, they were termed as the iHE sign (Figure 1B), an ultrasound finding previously described as specific for CPPD [16]. Figure 1 further clarifies the differentiation between the DC sign and the iHE sign. DoV was graded on a 0–3 scale with 0 having no Doppler signal, grade 1 demonstrating three or less isolated Doppler signals, grade 2 demonstrating three or more distinct Doppler signals, and grade 3 demonstrating multiple converging Doppler signals [17]. Ultrasound was performed prior to arthrocentesis; thus, the examiner was blinded to the final diagnosis.

### 2.3. Arthrocentesis

Only patients who underwent the reference standard of arthrocentesis with compensated polarized light microscopy were included in the final review. Microscopy was performed by a board-certified rheumatologist. Negatively birefringent needle-shaped crystals were classified as gout and positively birefringent rhomboid-shaped crystals as CPPD. If no crystals were visualized, diagnostic criteria for OA, such as the presence of osteophytes on ultrasound, were checked. Osteoarthritis was diagnosed based on ultrasound, arthrocentesis, and clinical presentation. A low leukocyte count in the joint aspirate (<0.2–2 cells/nL) with a majority lymphocyte/monocyte cell differentiation was indicative of OA [18]. Radiographs were not obtained.

### 2.4. Laboratory Parameters

Routinely determined laboratory parameters were used in the statistical analysis (laboratory-specific normal values in brackets): ferritin (40–300 ng/mL), uric acid (<7.0 mg/dL), ESR (3–8 mm/h), CRP (<5 mg/L), and leukocyte cell count of the aspirate (3.5–9.8 cells/nL).

### 2.5. Statistical Analysis

Analysis was performed using the R software environment (version 4.0.2; R Core Team, Vienna, Austria) by a trained statistician (C.B.) [19]. Continuous variables are described as mean and standard deviation and as median and interquartile range (IQR) where normality could not be assumed. Categorical variables are presented as absolute and relative frequencies. Unifactorial two-tailed analyses of variance (ANOVAs) were carried out for continuous variables with Scheffe’s post-hoc test. When normality could not be assumed, data was analyzed using the Mann–Whitney *U* test. Categorical data was analyzed using the chi-square test. The *p*-values are reported as part of descriptive analyses. Simple linear regression analyses were performed regarding age, sex, BMI, renal status, arterial hypertension, cumulative joint score, total number of affected joints, DC sign, iHE sign, DoV, serum uric acid concentrations, ESR, ferritin, and leukocyte cell count compared to the x-axis of CRP. Simple linear regressions were performed as complete case analyses. Additionally, conditional inference trees (Ctrees) were used to examine classification rules for gout, OA, and CPPD. This analysis recursively partitions the dataset to form subgroups that are as distinct as possible from each other. To partition the dataset, the cut-off value of an independent variable that provides the best partitioning is chosen. The procedure is repeated until no association with the outcome can be found in a permutation test at a given significance level of alpha = 0.05 [20]. The outcome was set to the diagnosis (OA, CPPD, or gout), confirmed via the respective reference standard. The independent variables included were sex, diabetic status, hypertension status, renal status, BMI, cumulative joint score, number of affected joints, DoV, DC sign, ferritin, uric acid, CRP, and leukocyte count. In case of missing data, the patient was counted to the right side of the splitting criterion. All relevant data is available within the manuscript.

## 3. Results

### 3.1. Patient Characteristics

**Figure 2:**
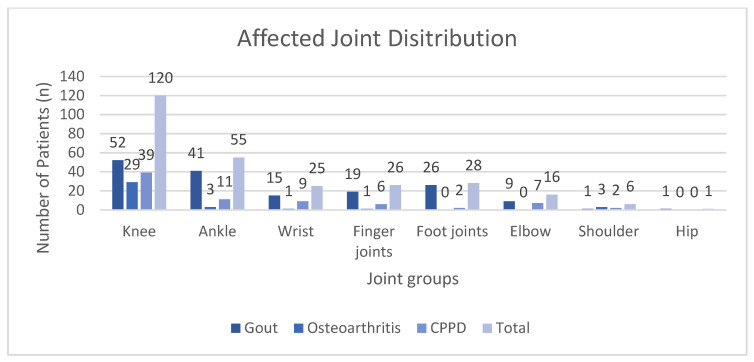
Distribution of the affected joints for each diagnosis grouped by joint categories. CPPD: calcium pyrophosphate deposition disease.

In total, 164 patients suffering from gout, 37 from OA, and 76 from CPPD were included into our study. Patient demographics are summarized in Table 2. Distribution of the affected joints is visualized in Figure 2.

### 3.2. Differentiation between Gout, CPPD, and OA by Biomarkers

Overall, 164 cases of gout (59.2%), 37 cases of OA (13.4%), and 76 cases of CPPD (27.4%) were included for analysis. Males comprised roughly 66% of patients, with as high as 76% in the gout group. The mean age of the OA group (64.5 years ± 17.3) was statistically significantly lower than that of the CPPD group (73.1 years ± 11.0), with Scheffe’s post-hoc *p*-value of 0.003. Approximately 43% of all analyzed joints were knees. Table 3 summarizes the results of the comparative statistical analysis.

**Figure 3:**
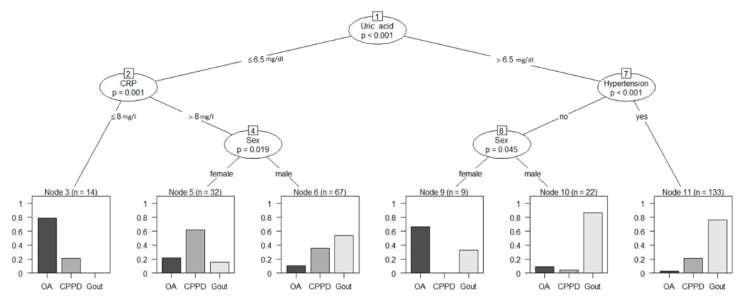
Conditional inference tree analysis for the classification of diagnoses based on clinical biomarkers. Reliable diagnostic predictions were possible using just the four biomarkers (uric acid, CRP, arterial hypertension, and sex) to differentiate gout (sensitivity 95.1%, specificity 41.6%), CPPD (sensitivity 26.3%, specificity 94.0%), and OA (sensitivity 46.0%, specificity 97.5%) in our patient population. Numbers above biomarkers represent node numbers. CRP: C-reactive protein; OA: osteoarthritis; CPPD: calcium pyrophosphate deposition disease.

Ctree analysis (Figure 3) was performed to further analyze the biomarkers and split the 277 patients according to their final diagnosis. When using Ctrees for prediction, each patient is assigned to the most frequent diagnosis in the respective end node (leaf). For this study population, a classification based on the conditional inference tree led to a sensitivity of 45.95% and specificity of 97.5% for OA, sensitivity of 26.32% and specificity of 94.03% for CPPD, and sensitivity of 95.12% and specificity of 41.59% for gout. Ctree sensitivity, specificity, positive predictive value, and negative predictive value are summarized in Table 4.

### 3.3. Association of Biomarkers with an Increased Inflammatory Response 

Simple linear regressions were performed to find out which biomarkers had the most prominent correlation with the inflammatory response as measured by elevated serum CRP and leukocyte count in the joint aspirate. Table 5 summarizes the results.

### 3.4. The Double Contour Sign as a Diagnostic Criterion for Gout

The DC sign on ultrasound was assessed regarding sensitivity and specificity for gout and CPPD. Out of 277 patients, 151 demonstrated a positive DC sign (54.4%), while 7 patients showed signs of an iHE (2.5%) and 16 had signs of both (5.8%). This resulted in a sensitivity and specificity of 71% and 55% for the DC sign in gout. If the DC sign was assumed to be diagnostic for CPPD, it achieved a sensitivity/specificity of 59%/39%. The iHE sign on the other hand demonstrated a high specificity of 99% but a sensitivity of only 26%. Comparative mean analysis (*t*-test) showed a significantly elevated serum uric acid (*p* = 0.02; mean 9.5 mg/L ± 3.7) in DC positive patients compared to DC negative patients (mean 8.1 mg/L ± 3.1).

## 4. Discussion

This study is the first to examine a large cohort of consecutive patients with acute arthralgia due to gout, CPPD, or OA in terms of regularly defined biomarkers identified through physical examination, laboratory tests, and imaging modalities such as ultrasound and polarized microscopy.

Gout affected significantly more males than either OA (76% vs. 38% respectively, *p* < 0.0001) or CPPD (76% vs. 58% respectively, *p* = 0.0043). The mean age of the gout group was 69.9 ± 11.9 years, which is comparable to a large study of German patients with an average age of 63.1 ± 13.1 years [21]. Interestingly, the OA group was significantly younger (64.5 ± 17.3 years) than the CPPD cohort (73.1 ± 11.0 years, *p* = 0.0031) but in line with the national average of 63.3 ± 8.8 years for knee and hip OA [22]. Unfortunately, there is a paucity of data regarding CPPD in the German population; yet, taking into account an American study, we discovered a comparable average age (73.1 ± 11.0 years vs. 68.1 ± 12.3 years) [23]. Unsurprisingly, gout patients also demonstrated statistically significant elevated serum uric acid when compared to OA (mean 9.1 vs. 5.6 mg/L, *p* < 0.0001) and CPPD (mean 9.1 vs. 6.7 mg/L, *p* < 0.0001), while no significant difference was observed between OA and CPPD (*p* = 0.28). The interplay between serum uric acid and hypertension has been described in numerous studies, with serum uric acid linked to increased cardiovascular mortality [24] and the development of hypertension [25,26]. Conversely, antihypertensive medication such as diuretics, beta-blockers, and alpha-1 blockers have been shown to reduce glomerular filtration and thus raise serum uric acid [27]. Unfortunately, due to the retrospective nature of this study, we were unable to explore the effects of current medication on serum uric acid levels, renal function, and hypertension. Furthermore, we were able to demonstrate an association between hypertension and gout in our study, with 81.7% of the patients in the gout group suffering from arterial hypertension (*n* = 134) compared to only 46% in the OA group (*n* = 17, *p* < 0.0001). These results correlate with a previous study from the USA, which demonstrated a high prevalence of 74% for hypertension in gout patients [28], and play an important role in the Ctree analysis to help differentiate gout from CPPD and OA. The rate of chronic kidney disease was also significantly higher in the gout cohort compared to the OA group (*p* < 0.0022), with a quarter of the gout group suffering from either stage 4 or 5 chronic kidney disease, compared to 15.8% in the CPPD group and 5.4% in the OA group, although no statistical difference was found between gout and CPPD (*p* = 0.21). The association between gout and chronic kidney disease has been the subject of many debates, but a definitive answer remains to be found [29]. Our work indirectly supports a link between gout and chronic kidney disease as inferred by descriptive statistics. Conflicting studies and meta-analyses have yet to come to a consensus, and a definitive pathomechanism eludes researchers to this day [30,31,32]. Gout affected significantly more joints than either OA (*n* = 2.2 vs. 1.4; *p* = 0.002) or CPPD (*n* = 2.2 vs. 1.6; *p* = 0.010), but it lacks clinical significance as the differentiation between 2.2 and 1.6 joints in a clinical setting has almost no application in real life scenarios. Cumulative joint score failed to show any significant difference between groups (ANOVA *p* = 0.07).

We were able to reliably differentiate between inflammatory and noninflammatory arthropathies based on the DoV during ultrasound examinations. Both gout and CPPD demonstrated a significant increase in the DoV on ultrasound vs. OA (both *p* < 0.0001). Power Doppler ultrasound was shown to demonstrate synovitis with a pooled sensitivity of 77.6% and specificity of 85.2% in a recent metanalysis [33]. As expected, gout and CPPD demonstrated markedly elevated ESR and CRP values. While patients in the OA group also had elevated levels of CRP and a high ESR value, they were statistically significantly lower than the two crystal arthropathies.

Using Ctree analyses, we were able to practically apply the above findings and create a flow chart that could have clinical applications, as demonstrated in Figure 3. Using recursive partitioning, we were able to predict gout with a positive predictive value of 0.70 while simultaneously excluding osteoarthritis (negative predictive value of 0.92) and CPPD (negative predictive value of 0.77) in our patient population using only four biomarkers: serum uric acid, sex, arterial hypertension, and serum CRP. This method could also serve further data analyses, thereby streamlining diagnostic processes by providing a list of differentials and their likelihoods to the attending physician for scrutiny after the input of commonly collected biomarkers.

On ultrasound, the DC sign was able to distinguish crystal arthropathies from OA (gout vs. OA, *p* < 0.0001; CPPD vs. OA, *p* < 0.0001) but could not reliably differentiate between gout and CPPD (*p* = 0.0762). Similar findings have been published in previous studies [17], which showed comparable results (sensitivity of 64% for gout and 52% for CPPD) to our observed sensitivity/specificity of 71%/55% for gout and 59%/39% for CPPD. The iHE sign had a remarkably high specificity of 99% for CPPD but a rather low sensitivity of 26% (*n* = 20 out of 76) and was only applicable in a few cases as the differentiation between paracartilaginous and intracartilaginous localization was not possible to a confident degree even for experienced sonographers.

Using simple linear regressions, people with type II diabetes, a higher average joint score, a greater number of affected joints, a positive DC sign on ultrasound, increased serum uric acid, and an elevated leukocyte count in the aspirate were seen to have higher CRP levels as a sign of an increased inflammatory response. Elevated CRP levels in type II diabetic patients have been the focus of many studies as it has been shown to be a risk factor for type II diabetes through low-grade inflammation [34,35,36,37,38], although the exact pathomechanism remains unknown. Both an increased joint burden and a higher cumulative joint score were correlated with an increased CRP response. Our joint score system has not yet been validated by other studies but aims to adjust the absolute number of involved joints for size. This approach is constrained by the fact that a larger joint is not always entirely inflamed and does not always elicit a response proportional to size. Few studies are currently available that explore the association of joint size involvement and CRP response in gout or CPPD. A study from 1987 [39] linked increased joint involvement to an increased CRP response in gout, while a more recent study was able to link a higher joint burden to an increased CRP reaction in female OA patients [40]. Similarly, a study from 2017 demonstrated a proportional CRP response based on joint size in rheumatoid arthritis patients [41] when compared to the same number of different sized joints. Current research suggests that serum uric acid contributes to elevated CRP levels. The pathomechanism of this response is thought to be due to binding of CRP to uric acid, leading to an activation of the complement system [42,43]. Uric acid has also been linked to an increased risk of hypertension [44], further complicating the relationship between uric acid, CRP, hypertension, renal function, and metabolic syndrome. To our knowledge, no recent papers have studied the association between a positive DC sign on ultrasound and CRP burden. The DC sign represents a visual confirmation of monosodium urate crystals and its subsequent effects on surrounding structures. An appreciable amount of crystal must be in the blood stream and already precipitated into juxta-articular tissue for its signal to be picked up on ultrasound. Comparative mean analysis confirmed significantly elevated serum uric acid (*p* = 0.02) in DC positive patients compared to DC negative patients. As a result, one might argue that the DC symbol is essentially a sonographic expression of elevated serum uric acid.

Our study has limitations due to its retrospective nature. Although Ctrees provided reliable predictive information regarding diagnosis, they only apply to the analyzed dataset. Our study was missing a healthy control group. Groups were also not of equal size. Further studies with larger cohorts and a control group are needed to further explore the utility of this tool. Patients presenting with oligoarthritis represent another limitation of our study as only the most symptomatic joints were aspirated. Theoretically, a non-aspirated joint might have been caused by a different pathology than the aspirated joint. 

## 5. Conclusions

We were able to predict gout in our patient population using four biomarkers (serum uric acid, CRP, sex, and arterial hypertension) with a predictive value of 0.70 and rule out OA (negative predictive value of 0.92) and CPPD (negative predictive value 0.77). The sonographic double contour sign was not able to reliably differentiate between gout and CPPD with a sensitivity and specificity of 71% and 55% for gout and 59%/39% for CPPD. The inflammatory response based on serum CRP levels correlated with patients suffering from type II diabetes, a higher cumulative joint score, an increased number of affected joints, positive DC sign on ultrasound, elevated serum uric acid, and elevated leukocyte count in the joint aspirate.

## Figures and Tables

**Table 1:** Cumulative joint score classification.

Classification	Joints	Points Per Joint
Small joints	DIP, PIP, MCP, MTP, or IP joints	1
Middle-sized joints	Ankle, elbow, or wrist joints	2
Large joints	Shoulder, hip, or knee joints	3

Joints were classified and allocated points based on their size. DIP: distal interphalangeal joints of the hands and feet; PIP: proximal interphalangeal joints of the hands and feet; MCP: metacarpophalangeal joints; MTP: metatarsophalangeal joints; IP: interphalangeal joints of the hallux or thumb.

**Table 2:** Patient characteristics.

Characteristic	Osteoarthritis	CPPD	Gout	Overall
	(*n* = 37)	(*n* = 76)	(*n* = 164)	(*n* = 277)
**Sex**				
Female, *n* (%)	23 (62.2%)	32 (42.1%)	39 (23.8%)	94 (33.9%)
Male, *n* (%)	14 (37.8%)	44 (57.9%)	125 (76.2%)	183 (66.1%)
**Age**				
Mean, years (SD)	64.5 (17.3)	73.1 (11.0)	69.9 (11.8)	70.1 (12.7)
Median [Min, Max]	64.4 [30.3, 93.0]	72.9 [39.0, 93.0]	72.1 [36.5, 90.0]	72.0 [30.3, 93.0]
**Diabetes**				
no, *n* (%)	30 (81.1%)	50 (65.8%)	106 (64.6%)	186 (67.1%)
yes, *n* (%)	7 (18.9%)	26 (34.2%)	58 (35.4%)	91 (32.9%)
**Weight**				
Mean, kg (SD)	89.4 (23.5)	81.1 (17.2)	85.8 (19.5)	85.0 (19.6)
Median [Min, Max]	84.0 [50.0, 127]	79.0 [38.0, 124]	83.0 [41.0, 168]	83.0 [38.0, 168]
Missing, *n* (%)	4 (10.8%)	6 (7.9%)	18 (11.0%)	28 (10.1%)
**Height**				
Mean, cm (SD)	171 (12.9)	169 (10.2)	171 (8.09)	170 (9.47)
Median [Min, Max]	170 [152, 198]	172 [140, 198]	172 [150, 187]	172 [140, 198]
Missing, *n* (%)	4 (10.8%)	6 (7.9%)	18 (11.0%)	28 (10.1%)
**Arterial hypertension**				
no, *n* (%)	20 (54.1%)	15 (19.7%)	30 (18.3%)	65 (23.5%)
yes, *n* (%)	17 (45.9%)	61 (80.3%)	134 (81.7%)	212 (76.5%)
**Renal function ^1^**				
Mean, stage (SD)	1.86 (0.976)	2.33 (1.17)	2.62 (1.21)	2.44 (1.19)
Median [Min, Max]	2.00 [1.00, 4.00]	2.00 [1.00, 5.00]	3.00 [1.00, 5.00]	2.00 [1.00, 5.00]
Stage 1, *n* (%)	18 (6.5%)	21 (7.6%)	42 (15.2%)	81 (29.2%)
Stage 2, *n* (%)	8 (2.9%)	26 (9.4%)	28 (10.1%)	62 (22.4%)
Stage 3, *n* (%)	9 (3.2%)	17 (6.1%)	53 (19.1%)	79 (28.5%)
Stage 4, *n* (%)	2 (0.7%)	7 (2.5%)	33 (11.9%)	42 (15.2%)
Stage 5, *n* (%)	0 (0%)	5 (1.8%)	8 (2.9%)	13 (4.7%)
**BMI**				
Mean, kg/m^2^ (SD)	30.4 (6.96)	28.2 (5.01)	29.3 (5.96)	29.1 (5.87)
Median [Min, Max]	30.0 [19.5, 47.4]	27.2 [18.8, 43.0]	28.7 [18.0, 61.0]	28.6 [18.0, 61.0]
Missing, *n* (%)	4 (10.8%)	6 (7.9%)	18 (11.0%)	28 (10.1%)
**Ferritin**				
Mean, ng/mL (SD)	428 (887)	351 (295)	353 (405)	361 (435)
Median [Min, Max]	68.0 [24.0, 2760]	241 [42.0, 1480]	161 [13.0, 1630]	224 [13.0, 2760]
Missing, *n* (%)	28 (75.7%)	38 (50.0%)	136 (82.9%)	202 (72.9%)
**Cumulative joint score ^2^**				
Mean, points (SD)	2.89 (0.567)	3.74 (3.11)	4.13 (3.18)	3.86 (2.97)
Median [Min, Max]	3.00 [1.00, 5.00]	3.00 [1.00, 15.0]	3.00 [1.00, 18.0]	3.00 [1.00, 18.0]
Missing, *n* (%)	0 (0%)	3 (3.9%)	0 (0%)	3 (1.1%)
**Number of affected joints**				
Mean, *n* (SD)	1.03 (0.164)	1.56 (1.40)	2.23 (1.77)	1.89 (1.61)
Median [Min, Max]	1.00 [1.00, 2.00]	1.00 [1.00, 8.00]	2.00 [1.00, 10.0]	1.00 [1.00, 10.0]
Missing, *n* (%)	0 (0%)	3 (3.9%)	0 (0%)	3 (1.1%)
**Double contour sign ^3^**				
no, *n* (%)	31 (83.8%)	31 (40.8%)	47 (28.7%)	109 (39.4%)
yes, *n* (%)	6 (16.2%)	45 (59.2%)	116 (70.7%)	167 (60.3%)
Missing, *n* (%)	0 (0%)	0 (0%)	1 (0.6%)	1 (0.4%)
**Intracartilaginous hyperechogenicity ^3^**				
no, *n* (%)	36 (97.3%)	56 (73.7%)	161 (98.2%)	253 (91.3%)
yes, *n* (%)	1 (2.7%)	20 (26.3%)	2 (1.2%)	23 (8.3%)
Missing, *n* (%)	0 (0%)	0 (0%)	1 (0.6%)	1 (0.4%)
**Degree of vascularization ^4^**				
Mean, stage *n* (SD)	0.811 (0.938)	1.79 (0.943)	2.04 (0.974)	1.80 (1.04)
Median [Min, Max]	1.00 [0, 3.00]	2.00 [0, 3.00]	2.00 [0, 3.00]	2.00 [0, 3.00]
Missing, *n* (%)	0 (0%)	0 (0%)	3 (1.8%)	3 (1.1%)
**Uric acid**				
Mean, mg/dL (SD)	5.60 (1.93)	6.74 (3.38)	9.12 (3.57)	8.05 (3.63)
Median [Min, Max]	5.25 [2.80, 11.8]	5.80 [2.20, 18.2]	8.60 [3.10, 21.8]	7.30 [2.20, 21.8]
Missing, *n* (%)	5 (13.5%)	2 (2.6%)	0 (0%)	7 (2.5%)
**Erythrocyte sedimentation rate**				
Mean, mm (SD)	23.0 (24.7)	55.8 (33.1)	65.6 (27.2)	55.7 (32.3)
Median [Min, Max]	17.0 [2.00, 120]	49.0 [7.00, 120]	67.0 [9.00, 120]	50.0 [2.00, 120]
Missing, *n* (%)	10 (27.0%)	26 (34.2%)	76 (46.3%)	112 (40.4%)
**CRP**				
Mean, mg/L (SD)	27.2 (44.1)	90.9 (75.1)	111 (84.2)	94.5 (82.2)
Median [Min, Max]	11.0 [1.00, 246]	75.0 [1.00, 283]	91.5 [2.00, 408]	74.0 [1.00, 408]
**Leukocyte count in aspirate**				
Mean, *n*/mL (SD)	0.484 (0.617)	19.8 (30.4)	25.1 (53.1)	18.7 (41.9)
Median [Min, Max]	0.300 [0, 2.90]	6.25 [0.0600, 150]	9.42 [0.170, 455]	5.71 [0, 455]
Missing, *n* (%)	5 (13.5%)	24 (31.6%)	82 (50.0%)	111 (40.1%)

CPPD: calcium pyrophosphate deposition disease; BMI: body mass index; CRP: C-reactive protein. ^1^ Renal status: classified according to the Kidney Disease Improving Global Outcome (K-DIGO) guidelines based on the glomerular filtration rate (GFR): 1 = GFR >90 mL/min, 2 = GFR 89–60 mL/min, 3 = GFR 59–30 mL/min, 4 = GFR 29–15 mL/min, and 5 = GFR <15 mL/min. ^2^ The cumulative joint score assigns points to each affected joint based on size. Small joints such as fingers and toes receive one point; middle-sized joints such as the wrist, ankle, or elbow get two points; and large joints such as the shoulder, knee, or hip receive three points. ^3^ The double contour sign was defined as hyperechoic bands adjacent to hyaline cartilage. Hyperechoic signals that could be confidently placed inside the cartilage were termed as an intracartilaginous hyperechogenicity specific for CPPD.^4^ Degree of vascularization was graded on a 0 to 3 scale, with 0 showing no observable increased vascularization, grade 1: three or less isolated Doppler signals, grade 2: three or more distinct Doppler signals, and Grade 3: multiple converging Doppler signals.

**Table 3:** Results of comparative statistical analysis of commonly derived clinical, laboratory, and imaging characteristics.

*p* Values/Characteristics	Gout	OA	CPPD	Gout *n*	OA *n*	CPPD *n*	Gout vs. CPPD	Gout vs. OA	OA vs. CPPD
Sex, female, *n* (%) *	39 (23.8)	23 (62.2)	32 (42.1)	164	37	76	0.0043	<0.0001	0.0704
Age, years (SD) ^+^	69.9 (11.9)	64.5 (17.3)	73.1 (11.0)	164	37	76	0.1950	0.0573	0.0031
Diabetes, *n* (%) *	58 (35.4)	7 (18.9)	26 (34.2)	164	37	76	0.8854	0.0786	0.1234
Weight, kg (SD) *	85.5 (19.5)	89.4 (23.5)	81.1 (17.2)	146	33	70	0.2417	0.6342	0.1282
Height, cm (SD) *	171.0 (8.1)	171.2 (12.9)	169.1 (10.3)	146	33	70	0.3840	0.9947	0.5777
Hypertension, *n* (%) ^+^	134 (81.7)	17 (46.0)	61 (80.3)	164	37	76	0.8592	<0.0001	0.0003
Renal function, severity score, mean (SD) ^1^	2.6 (1.2)	1.9 (1.0)	2.3 (1.2)	164	37	76	0.2107	0.0022	0.1423
BMI, kg/m^2^ (SD) *	29.3 (6.0)	28.9 (5.8)	28.2 (5.0)	146	33	70	0.4815	0.6002	0.2157
Ferritin, ng/mL (SD) *	352.5 (405.3)	427.9 (886.8)	350.7 (294.7)	28	9	38	0.9999	0.9050	0.8942
Cumulative joint point score, mean (SD) *^,2^	4.1 (3.2)	2.9 (0.6)	3.7 (3.1)	164	37	73	0.6457	0.0723	0.3637
Number of affected joints, *n* (SD) *	2.2 (1.8)	1.0 (0.2)	1.6 (1.4)	164	37	73	0.0099	0.0002	0.2355
Double contour sign ^3^, *n* (%) ^+^	116 (70.7)	6 (16.2)	45 (59.2)	163	37	76	0.0762	<0.0001	<0.0001
Intracartilaginous hyperechogenicity ^3^, *n* (%) ^+^	2 (1.2)	1 (2.7)	20 (26.3)	163	37	76	<0.0001	0.4954	0.0036
Degree of vascularisation, degree ^4^, mean (SD) *	2.0 (1.0)	0.8 (0.9)	1.8 (0.9)	161	76	37	0.1814	<0.0001	<0.0001
Uric acid, mg/dL (SD) *	9.1 (3.6)	5.6 (1.9)	6.7 (3.4)	164	74	32	<0.0001	<0.0001	0.2799
Erythrocyte sedimentation rate, mm (SD) *	65.7 (27.2)	23.0 (24.7)	55.8 (33.1)	88	27	50	0.1574	<0.0001	<0.0001
C-reactive protein, mg/L (SD) *	111.4 (84.2)	27.2 (44.1)	90.9 (75.1)	164	37	76	0.1657	<0.0001	0.0003
Leukocyte count in aspirate, n/nL (SD) *	25.1 (53.1)	0.5 (0.6)	19.8 (30.4)	82	32	52	0.7706	0.0181	0.1157

Summary of ANOVA with Scheffe’s test * or Mann–Whitney *U* tests ^+^. *p*-values of less than 0.05 were deemed statistically significant. All values are reported as mean unless otherwise noted. OA: osteoarthritis; CPPD: calcium pyrophosphate deposition disease; BMI: body mass index. ^1^ Renal function: classified according to the Kidney Disease Improving Global Outcome (K-DIGO) guidelines based on the glomerular filtration rate (GFR): 1 = GFR > 90 mL/min, 2 = GFR 89–60 mL/min, 3 = GFR 59–30 mL/min, 4 = GFR 29–15 mL/min, and 5 = GFR < 15 mL/min. ^2^ The cumulative joint score assigns points to each affected joint based on size. Small joints such as fingers and toes receive one point; middle-sized joints such as the wrist, ankle, or elbow get two points; and large joints such as the shoulder, knee, or hip receive three points. ^3^ The double contour sign was defined as hyperechoic bands adjacent to hyaline cartilage. Hyperechoic signals that could be confidently placed inside the cartilage were termed as an intracartilaginous hyperechogenicity specific for CPPD. ^4^ Degree of vascularization was graded on a 0 to 3 scale, with 0 showing no observable increased vascularization, grade 1: three or less isolated Doppler signals, grade 2: three or more distinct Doppler signals, and grade 3: multiple converging Doppler signals.

**Table 4:** Diagnostic performance of conditional inference tree analysis.

	Gout	CPPD	OA
Sensitivity	95.1%	26.3%	46.0%
Specificity	41.6%	94.0%	97.5%
PPV	0.703	0.625	0.739
NPV	0.855	0.771	0.921

CPPD: Calcium pyrophosphate deposition disease; OA: osteoarthritis; PPV: positive predictive value; NPV: negative predictive value.

**Table 5:** Simple linear regression models for C-reactive protein (CRP) and leukocyte count in the aspirate versus biomarkers.

Independent Variable	CRP			Leukocyte Count		
	Intercept	Beta	*p*-Value	Intercept	Beta	*p*-Value
Sex (male)	90.48	6.09	0.56	23.71	−7.60	0.2704
Age	81.93	0.18	0.6461	0.40	0.26	0.259
Diabetes	87.67	20.79	0.04785	18.19	1.46	0.833
Hypertension	87.70	8.89	0.4465	12.95	7.98	0.2699
Renal status	84.42	4.14	0.3196	20.55	−0.79	0.7726
BMI	82.61	0.42	0.6455	9.20	0.34	0.6044
Ferritin	85.46	0.02	0.3395	37.47	−0.03	0.2678
Cumulative joint size	66.14	7.29	1.05 × 10^−5^	18.75	<0.00	0.9992
Number of affected joints	67.60	14.09	3.51 × 10^−6^	18.42	0.17	0.9323
Double contour sign	76.37	29.33	0.00599	20.45	−3.41	0.703
Intracartilaginous hyperechogenicity sign	94.11	0.07	0.4253	18.98	−4.57	0.7338
Degree of vascularization	81.69	6.61	0.1667	15.82	1.86	0.5644
Serum uric acid	72.38	2.87	0.0381	17.01	0.28	0.7722
ERS	15.13	1.25	9.97 × 10^−12^	6.05	0.25	0.08,273
CRP	-	-	-	1.35	0.18	2.07 × 10^−6^
Leukocyte count	82.72	0.71	2.07 × 10^−6^	-	-	-
Diagnosis CPPD	27.16	63.73	5.48 × 10^−5^	0.48	19.31	0.03810
Diagnosis gout	27.16	84.21	7.54 × 10^−9^	0.48	24.58	0.00467

Complete cases analyses were carried out for serum CRP and leukocyte count of the aspirate as the outcome with the biomarkers as independent variables. *p*-values less than 0.05 were considered significant for descriptive statistical analysis. BMI: body mass index; ERS: erythrocyte sedimentation rate; CPPD: calcium pyrophosphate deposition disease.

## Data Availability

Raw data is available upon request.
